# Human-like perceptual masking is difficult to observe in rats performing an orientation discrimination task

**DOI:** 10.1371/journal.pone.0207179

**Published:** 2018-11-21

**Authors:** Katrina Louise Dell, Ehsan Arabzadeh, Nicholas Seow Chiang Price

**Affiliations:** 1 Neuroscience Program, Biomedicine Discovery Institute, Monash University, Clayton, VIC, Australia; 2 Department of Physiology, Monash University, Clayton, VIC, Australia; 3 Australian Research Council Centre of Excellence for Integrative Brain Function, Monash University Node, Clayton, VIC, Australia; 4 Department of Medicine, The University of Melbourne, St. Vincent’s Hospital, Fitzroy VIC, Australia; 5 Eccles Institute of Neuroscience, John Curtin School of Medical Research, The Australian National University, Canberra, ACT, Australia; 6 Australian Research Council Centre of Excellence for Integrative Brain Function, The Australian National University Node, Canberra, ACT, Australia; State University of New York Downstate Medical Center, UNITED STATES

## Abstract

Visual masking occurs when the perception of a brief target stimulus is affected by a preceding or succeeding mask. The uncoupling of the target and its perception allows an opportunity to investigate the neuronal mechanisms involved in sensory representation and visual perception. To determine whether rats are a suitable model for subsequent studies of the neuronal basis of visual masking, we first demonstrated that decoding of neuronal responses recorded in the primary visual cortex (V1) of anaesthetized rats predicted that orientation discrimination performance should decline when masking stimuli are presented immediately before or after oriented target stimuli. We then trained Long-Evans rats (n = 7) to discriminate between horizontal and vertical target Gabors or gratings. In some trials, a plaid mask was presented at varying stimulus onset asynchronies (SOAs) relative to the target. Spatially, the masks were presented either overlapping or surrounding the target location. In the absence of a mask, all animals could reliably discriminate orientation when stimulus durations were 16 ms or longer. In the presence of a mask, discrimination performance was impaired, but did not systematically vary with SOA as is typical of visual masking. In humans performing a similar task, we found visual masking impaired perception of the target at short SOAs regardless of the spatial or temporal configuration of stimuli. Our findings indicate that visual masking may be difficult to observe in rats as the stimulus parameters necessary to quantify masking will make the task so difficult that it prevents robust measurement of psychophysical performance. Thus, our results suggest that rats may not be an ideal model to investigate the effects of visual masking on perception.

## Introduction

The perception of a stimulus is altered by the context in which it is presented. In visual masking, the perception of a brief target stimulus is impaired by a mask presented in close spatial and temporal proximity [1]. The neuronal mechanisms responsible for this phenomenon remain unclear, but are likely to involve interactions throughout the visual processing hierarchy starting as early as the retina [2]. Varying the temporal separation between target and mask stimuli by just a few milliseconds systematically alters the perception and neuronal representation of the target, providing the opportunity to investigate the neuronal mechanisms involved in the development of conscious visual perception.

Masking effects depend strongly on the temporal and spatial configuration of the target and mask stimuli [3, 4]. The temporal categories of visual masking include forward and backward masking, which describe when target perception is impaired by a mask that precedes or succeeds the target stimulus in time, respectively [5]. Backward masking is of particular interest as target perception is retroactively reduced by mask-evoked neuronal activity and therefore cannot be explained by adaptation in the early sensory processing pathway [6]. The spatial categories of visual masking are broadly divided according to whether the mask overlaps the target’s location or is presented in a non-overlapping location, typically as a surround with contiguous contours [5]. Centre-surround masking is commonly used because the size and relative position of the stimuli means that any neural interaction between them must occur primarily in the cortex [7]. Psychophysically, visibility of a target presented to one eye may be reduced by a mask presented to the other eye [8, 9], suggesting that perceptual masking involves binocular interactions which do not occur until the primary visual cortex (V1) [10].

Although masking is widely used in human perceptual studies, investigating the associated neuronal mechanisms necessitates an animal model [1]. Of the studies that have recorded neuronal responses to visual masking stimuli [11–23], only a handful have characterized both the neuronal and psychophysical effects within the same species, and only three studies collected neuronal and perceptual data simultaneously [12, 14, 16]. In order to address this gap, we aim to develop a model that will allow alert rats to report what they perceive, simultaneous to neuronal data collection.

Despite their impoverished spatial acuity and contrast sensitivity [24–26], rats are increasingly popular in vision research because they offer the capacity to collect data from large cohorts coupled with good options for genetic manipulations [27, 28]. Furthermore, anatomical and functional studies indicate rats possess visual processing pathways specialised for processing shape and motion information, like the ventral and dorsal pathway in primates [29–32]. Importantly, rodent detection and discrimination performance on a variety of tasks is comparable to primates [24, 25, 33–36]. Our electrophysiological studies of masking in anesthetized rats showed that neuronal responses in V1 to oriented targets were altered by spatially overlapping and surround masks [2]. However, it remains to be seen whether these changes are accompanied by perceptual deficits, or if visual masking in rats and humans follow similar trends. If perceptual masking is not readily observed in rats, the benefits of a combined perceptual and neuronal study would be limited.

We first predicted rat perceptual performance on an orientation discrimination task by linearly decoding neuronal responses to masking stimuli. Subsequently, we tested rats in three orientation discrimination tasks. In experiment 1, we varied target duration to assess the temporal acuity of rats. In experiment 2, we varied the SOA between target and mask to ascertain the influence of a spatially overlapping mask on discrimination performance and response times. In experiment 3, we further assessed the effects of backward masking on discrimination performance using both spatially overlapping and surround masks. Finally, we collected perceptual data from humans using similar stimuli to those used in the rat tasks. While our neuronal decoding predicts that perceptual performance should decline as SOA is reduced, and human perception was strongly affected by masking stimuli, we found little evidence of perceptual masking in rats. Altogether, our results suggest that essential aspects of visual masking, such as the inclusion of multiple, briefly presented stimuli, limit the performance of rats and thus our ability to observe perceptual masking in rats.

## Methods

All experimental procedures involving animals were approved by the Monash University Committee for Ethics in Animal Experimentation (MARP/2013/81; MARP/2013/130) and were conducted in accordance with the National Health and Medical Research Council guidelines for the care and welfare of experimental animals. All experimental procedures involving humans were approved by the Monash University Human Research Ethics Committee (CF16/392–2016000178) and were conducted in accordance with the National Statement on Ethical Conduct in Human Research. Participants gave written, informed consent prior to data collection.

### Neuronal decoding

Predictions of rats’ ability to discriminate vertical and horizontal oriented targets were generated by decoding previously published neuronal data, collected from extracellular recordings in V1 of 37 halothane-anaesthetised Long-Evans rats [2]. While orientation selectivity is preserved under halothane anaesthesia [37], the absence of higher-order feedback and perceptually-related signals should only impair our ability to predict perceptually-related masking effects from the neuronal data. Neuronal responses to spatially overlapping and centre-surround visual masking stimuli were recorded. Target stimuli were square-wave gratings (12 orientations; 4 phases) presented in a circular aperture matched to the receptive field of the neurons. The masks were black and white hyperplaids generated randomly on each trial by binarising the sum of gratings with all 12 possible target orientations and random phase. Mask stimuli were either presented at the same spatial location and dimensions as the target (spatially overlapping) or were presented full-screen with an aperture matching the target size and location (centre-surround). All stimuli were presented for 33.3 ms, with either 10 SOAs (±33.3–333.3) for the spatially overlapping condition or 16 SOAs (±8.3–333.3) for the centre-surround condition. When no target or mask was visible, a blank gray screen was displayed (luminance = 53.2 cd/m^2^). Only neurons that were tuned to orientation (d’_pref vs null_> 0.3) were included in the decoding analyses. Due to limitations in data collection, the number of orientation-selective neurons was different for the spatially-overlapping (SO) and centre-surround (CS) conditions, and for Forward and Backward masking: n_SO_Forward_ = 73; n_SO_Backward_ = 95; n_CS_Forward_ = 42; n_CS_Backward_ = 63. Here, we focus only on responses to horizontal and vertical target gratings, and the responses to gratings with the preferred and null orientation of the neuron.

We used spike counting windows from 50–100, 50–150 and 50–300 ms after target appearance. While the overall level of decoding performance differed depending on time window, the effects of SOA on performance were qualitatively similar. We therefore focussed on the 50–300 ms window, as it encompassed the entire neuronal response to the target. As neurons were not recorded simultaneously, to simulate population responses we generated 200 pseudo-populations each containing 20 neurons sampled without replacement from our database of neurons. For each pseudo-population, we simulated 10,000 trials of horizontal and vertical target gratings (and preferred and null targets), by drawing spiking responses from each neuron, with replacement. Unlike the horizontal and vertical comparison, the preferred and null orientations were different for each neuron, but in effect this does not matter because neurons were already recorded independently. Each neuron therefore contributes to two decoders: a horizontal-versus-vertical decoder, in which many neurons may be relatively uninformative; and a preferred-versus-null decoder, in which each neuron is maximally informative when considered independently. The preferred-versus-null decoder allowed us to look at the effect of masking on neuronal responses where discriminability was at its best across the population. Each neuron was tested with at least 32 repetitions of each orientation and SOA, meaning that there are at least 32^20^ possible pseudo-trials that can be created by combining the responses of each neuron to a particular stimulus. We then used Fisher’s linear discriminant analysis to predict whether the presented stimulus was horizontal or vertical. Separate trials were used for training and testing, and decoding performance was 10-fold cross-validated. Decoders were trained and tested separately for each SOA, temporal category and spatial category of masking.

### Rat perception

Data were collected from seven adult male rats weighing 300-400g. Long-Evans rats were selected for their high visual acuity (~1.0 cycle/degree) [26]. Rats were group-housed in environmentally enriched enclosures with a 12:12 hr reversed light-dark cycle. Animals had *ad libitum* access to food, but daily water consumption was restricted to rewards obtained during experimentation as well as a two-hour period of access following the last test session in a day. Test sessions were run once or twice daily, five days/week. On non-testing days, animals had *ad libitum* access to water.

Three rats were excluded from a cohort of ten as their performance remained at chance during the initial phase of orientation discrimination training.

#### Testing apparatus

Training was conducted in a custom Plexiglas testing chamber (20W x 30L x 40H cm) with three infrared photo-interrupters (Little Bird Electronics, GP1A57HRJ00F) embedded in the front ‘viewing’ wall of the enclosure. Rats activated the sensors by performing a ‘nose-poke’, blocking the infrared photo-interrupter beam with their nose. Animals used the central sensor to initiate stimulus presentation and the two flanking sensors to indicate their perceptual response. The flanking sensors incorporated a 16-gauge stainless steel tube for reward delivery from a computer-controlled syringe pump (New Era Pump Systems, NE-500). Visual stimuli were presented to rats on 120 Hz LCD monitors (Samsung 2232RZ or Eizo FG2421)[38] positioned 25 cm from the viewing wall. All stimuli were generated in MATLAB, using the Psychophysics Toolbox extensions [39–41]. Photo-interrupter outputs were sampled at 120 Hz (Measurement Computing, USB 1208FS) by custom MATLAB scripts, which also registered rat behaviour, controlled stimulus presentation and administered rewards or timeouts.

#### Rat discrimination task

Rats performed a two-alternative forced choice (2AFC) orientation discrimination task, with visual stimuli presented in one of three trial structures: 1) target only; 2) variable-duration; or 3) fixed-duration. Each trial structure was tested in a discrete block over a number of consecutive sessions. All stimuli were presented against a gray background (mean luminance: Samsung– 113 cd/m2; Eizo—79 cd/m2). At the end of stimulus presentation on each trial a 3.3 kHz ‘trial complete’ tone signalled to the rats that they could leave the central port and indicate their perceived target orientation at a flanking port. Each target orientation was assigned to a single flanking response sensor for the duration of the study. Correct responses were rewarded with 0.05–0.075 ml of 5% sucrose solution. Incorrect responses received no reward and incurred a 3–6 second timeout period, delaying the possible onset of the next trial. Trials in which rats left the central sensor before the tone were not rewarded.

To prevent reward port bias, if 3 consecutive incorrect choices for the same orientation were made, a ‘correction trial’ was implemented, in which the target was fixed to that orientation until a correct response was obtained. Correction trials were excluded from analyses. In practice, after ~21 days of performing the task, correction trials were rarely needed.

#### Experiment 1- target only trials

The influence of target duration on orientation discrimination performance was examined for all rats (n = 7). Target stimuli were full contrast (100%) Gabors with orientation 0 or 90°, spatial frequency 0.1 cycles/degree and random phase. The space constant, defined as the standard deviation of the Gaussian applied to the contrast envelope, was adjusted between 6–18° (average of 14°). The stimulus size was adjusted according to each rat’s performance, so that all rats were performing the task with the smallest stimulus size at which they could correctly discriminate orientation 70% of the time. Target stimuli were presented for 8.3–100 ms within a fixed trial duration of 1000 ms. To prevent impulsive behaviour in the animals, each trial included randomized pre-target (350–650 ms) and post-target (183–633 ms) hold periods.

#### Experiment 2- variable-duration trials

Three animals (A, B and C) were trained to complete sessions with forward and backward masking trials randomly interspersed. This ensured that the motivational state of the rats remained consistent across both forward and backward masking conditions. Target stimuli were identical to those used in target-only trials and the mask was a spatially overlapping plaid created by summing both target stimuli. Target stimuli had 100% contrast while mask stimuli had either 20 or 40% contrast. In each trial, the target and mask were presented at one of 13 stimulus onset asynchronies (SOA; -250 to 250 ms), where negative SOAs indicate forward, and positive indicate backward masking trials. We selected to use both a 16 ms and a 42 ms target duration, which was in trade-off between maintaining overall performance levels, while retaining a target duration that would enable masking to occur. In the 42 ms target condition trials with short SOAs (±16, 33 ms) the presentation of the target was cut-off by the presentation of the mask, while for SOA of 0, only the mask was visible. The mask was always presented for 42 ms. Trial duration varied between 316–650 ms, with the trial ending at the completion of the second stimulus; the mask in backward masking trials and the target in forward masking trials. Thus, trial duration was correlated with SOA. To investigate how trial duration variability might influence rat behaviour we measured response times from the onset of the target and mask. Animals had 8.3 s to leave the central nosepoke and a further 16.6 s to choose a response port, as such, there was no penalty for long response times and no explicit incentive for short response times. Aborted trials, where the animal left the central nosepoke before the tone, were not rewarded. Trials where response times were implausibly short (<100 ms) were excluded from percent correct analyses.

#### Experiment 3- fixed-duration trials

Three animals (D, E and F) were trained to complete masking sessions with randomly interspersed backward masking and control trials, which only included a target stimulus. We implemented two spatial configurations of stimuli in separate data collection blocks. In the spatially overlapping block, stimuli were the same as in the variable duration trials but with a 100% contrast mask. In control trials, the target was presented for the same duration that would have occurred at each SOA under masked conditions (16, 24, 33, & 42 ms). This allowed us to separate the effects of masking from stimulus duration at short SOAs under spatially overlapping conditions. In addition, we collected masking data using stimuli that did not spatially overlap. In the spatially distinct block, we used a centre-surround arrangement allowing us to examine the effects of a mask on target discrimination at short SOAs, without any changes in target duration. For the centre-surround condition, target stimuli were circular sine-wave gratings 22° in diameter with a spatial frequency of 0.1 cycles/degree. Mask stimuli were full screen plaids with an aperture matching the target size and location.

On each trial, the pre-stimulus delay, target orientation and SOA (in masked trials) were randomly selected. In masked trials, a mask stimulus was presented at one of 8 SOAs (16–250 ms) following the target. Regardless of SOA, all trials were 750 ms to prevent impulsive behaviour. After 750 ms, a ‘trial complete’ tone sounded and animals were allowed to leave the central nosepoke and make their choice.

### Human discrimination task

Two authors and four naïve subjects took part in the experiments. All participants had normal or corrected to normal vision. Each subject performed a training session for both spatially overlapping and centre-surround stimulus types.

Visual stimuli were generated using Psychtoolbox in MATLAB and were presented on an 85 Hz refresh rate CRT monitor positioned at a viewing distance of 50 cm. Target and mask stimuli were presented in both spatially overlapping and centre-surround spatial arrangements. For the spatially overlapping condition, the fixation point and visual stimuli were presented in the centre of the monitor. Target stimuli were Gabors with orientation 0 or 90°, spatial frequency 0.2 cpd and space constant 3°. Mask stimuli were a plaid generated by the sum of both target stimuli. For the centre-surround condition, target stimuli were circular sine-wave gratings with a 7° diameter. Relative to the centre of the screen, the fixation point was positioned 10° to the left, while target stimuli were centred 10° to the right. Mask stimuli were a full-screen grating with an aperture matching the size and location of the target. In both spatial conditions, mask stimuli were 100% contrast and 47 ms in duration, with SOAs of ±11–165 ms relative to the target. Target stimuli were presented at 10, 20, 30 and 100% contrast with duration 47 ms (spatial overlapping) or 12 ms (centre-surround). Under spatially overlapping conditions, six SOAs (± 11, 24, 35 ms) were temporally overlapping meaning that presentation of the target was cut off by the mask.

Head position was stabilized with a chin rest. A total of ~2400 trials/subject were collected over four data collection sessions (2 spatially overlapping and 2 centre-surround sessions). Within a session, trials were presented in blocks of 50, allowing participants to take frequent breaks if needed. On each trial, participants fixated on a small cross before a target and mask were presented and then indicated their perceived target orientation by button press. Correct discriminations were indicated by a brief tone. Response times were measured from the onset of the target stimulus until the key press response. Participants were allowed to respond at any time after the target onset but were not explicitly requested to respond as quickly as possible for the task. Trials extended until a response was made, so the only incentive to respond quickly was to begin another trial.

## Results

### Neuronal decoding predicts impaired discrimination performance at short stimulus onset asynchronies

We have previously demonstrated that single V1 neurons in anaesthetized rats are orientation tuned for static grating stimuli presented for just 33 ms, but that the presence of mask stimuli at short SOAs decreases neuronal orientation selectivity [2]. Here we apply linear decoding methods to populations of neurons in order to predict how masks with varying SOA might affect the animal’s ability to perceptually discriminate horizontal and vertical orientations.

**Fig 1:**
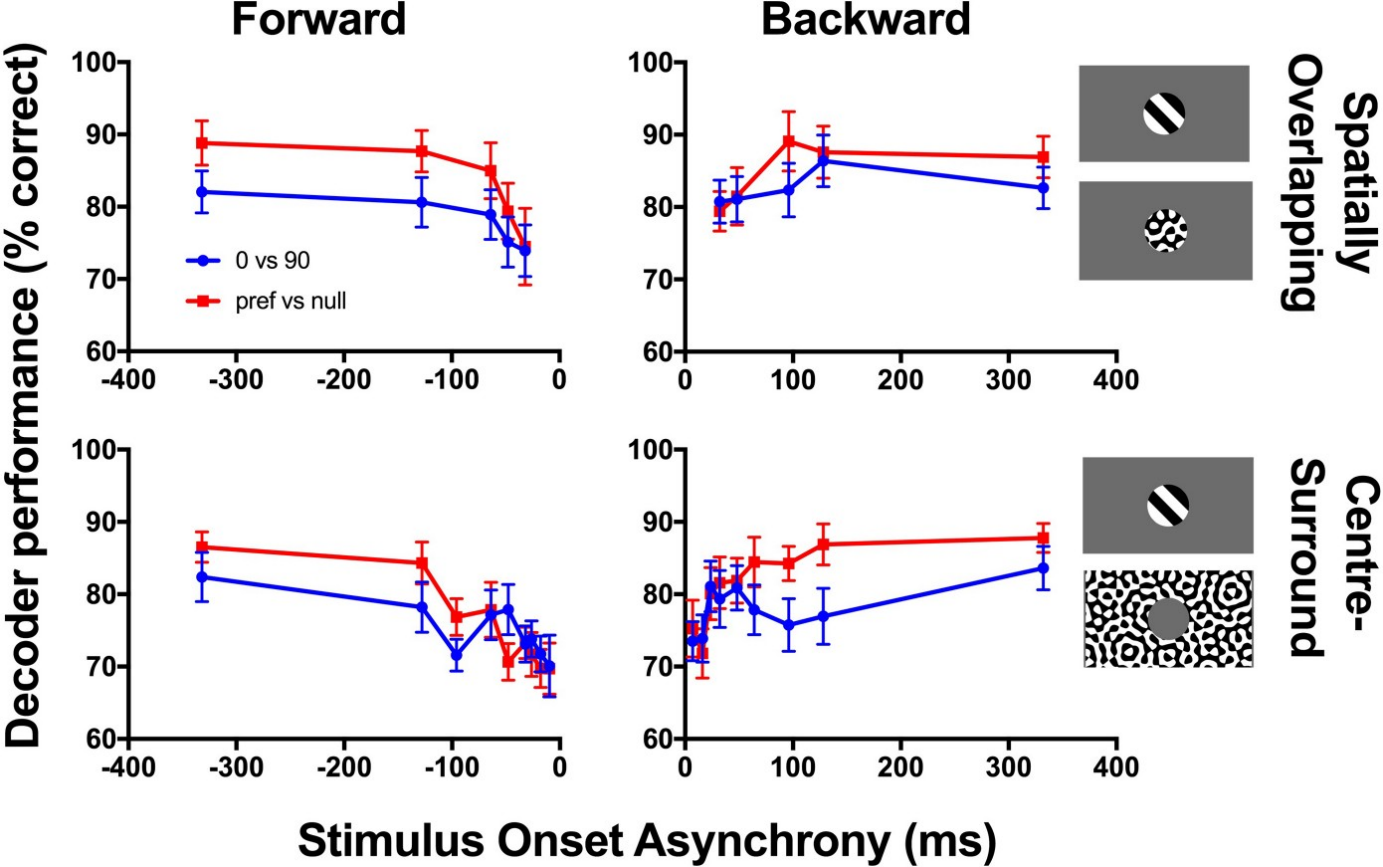
Decoding of V1 neuronal responses predicts that perceptual discrimination of orientation will decrease as stimulus onset asynchrony (SOA) approaches zero. We generated 200 pseudopopulations of 20 neurons, selected randomly without replacement. For each pseudopopulation we simulated 10,000 trials of horizontal and vertical target gratings (blue) or gratings with preferred and null orientations (red). We used Fischer’s linear discriminant to predict the stimulus orientation. The decoder was trained and tested separately for each spatial condition and for each SOA. Error bars indicate standard deviation across the 200 decoded pseudopopulations.

As our neurons were not recorded simultaneously, we generated 200 pseudo-populations of 20 neurons, drawing neurons without replacement from our neuronal database. For each SOA and masking condition, we then decoded the responses to 10,000 trials to predict whether the stimulus was a vertical or horizontal grating. We followed the same procedures for neuronal responses to the preferred and null orientations. With large population sizes (i.e. 100 or more neurons), our decoders perform close to 100% correct, because each neuron carries information about orientation that is nearly independent from the information available from other neurons. As we are interested here in how SOA manipulations affect decoding performance, we therefore restricted our populations to include only 20 neurons. We focus on spikes counted in the window 50–300 ms after target appearance, but shorter spike counting windows (50–100 ms; 50–150 ms) produced similar trends. The aim here was to compare relative decoding performance as SOA was varied. In general, the neuronal data predicted a drop in performance towards shorter SOAs regardless of the temporal (forward versus backward masking) or spatial layout of stimuli (spatially overlapping versus centre-surround; Fig 1). For spatially overlapping masking, the drop in performance was always monotonic and was greater for forward masking conditions, reflecting the same trends that are seen in psychophysical performance for spatially overlapping stimuli [42]. For centre-surround stimuli, the drop in performance towards shorter SOAs was not strictly monotonic, with a local minima occurring at an SOA of 100 ms for both forward and backward masking conditions. This may reflect an amalgamation of both the monotonic and U-shaped psychophysical trends that can be observed with centre-surround stimuli.

### Experiment 1: Rats can discriminate the orientation of brief target stimuli

**Fig 2:**
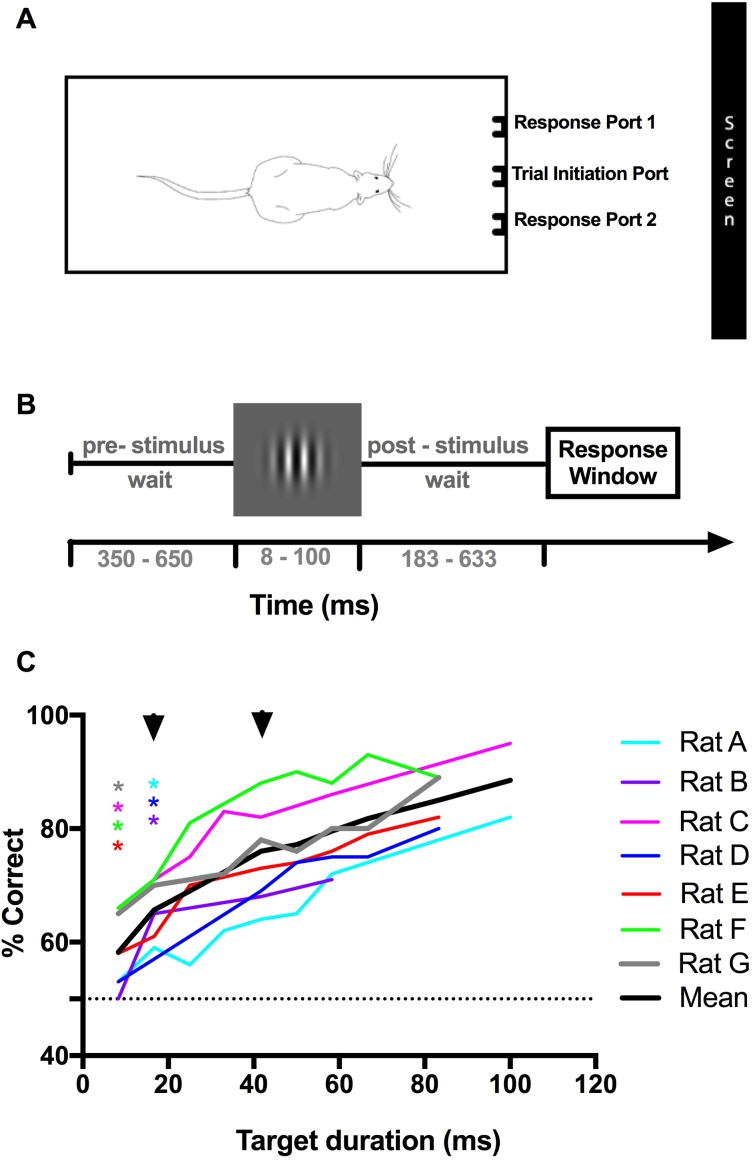
Rat orientation discrimination performance increases with target stimulus duration. (A) The testing chamber is fitted with three photo-interrupter detectors; a central sensor for trial initiation and two flanking sensors equipped with drinking spouts for perceptual report. The rat activates each sensor by breaking the infrared beam with its nose. Trial initiation results in the presentation of visual stimuli on an LCD monitor placed at a 25 cm viewing distance. When the rat selects the correct response port, a liquid reward is delivered at the centre of the corresponding flanking sensor. (B) Trial structure. Rat performance was measured in the absence of a mask across varying target durations. Following a central nose-poke, a blank gray screen was shown for a random period of 350–650 ms. Subsequently, the target grating was visible for 8.3–100 ms, followed by another random period with blank screen. After this period, a tone sounded, signalling that animals could leave the central port and move to a side port to indicate their response. Correct responses were only rewarded if they were made in the allocated response window. (C) Individual and average performance across 7 animals. The dotted line indicates chance performance. For each rat, the target duration where performance became significantly above chance is indicated by an asterisk (*, p<0.05). The black arrows indicate the target duration (16 and 42 ms) selected for subsequent masking experiments. For rat A, data represents a summary of performance across an average of 155 trials/duration and 17 sessions (Rat B– 214 trials/duration; 9 sessions. Rat C– 298 trials/duration; 24 sessions. Rat D– 327 trials/duration; 23 sessions. Rat E– 381 trials/duration; 25 sessions. Rat F– 191 trials/duration; 9 sessions. Rat G– 226 trials/duration; 29 sessions).

Visual stimuli are more easily masked if they are presented briefly, but even in the absence of a mask, a stimulus that is presented for too short a time will be difficult to detect. Therefore, studying masking requires finding a target duration that is well-perceived on its own, but still able to be masked. As no studies have previously examined the temporal limits of rat vision, we first examined how stimulus duration affected orientation discrimination of high contrast targets. Seven male Long-Evans rats were trained to perform a two-alternative forced choice discrimination between vertical and horizontal target Gabors presented for 8.3–100 ms (Fig 2A and 2B). For four rats (C, E, F & G) performance was significantly above chance for all target durations; the remaining rats (A, B & D) performed significantly above chance for target durations of 16 ms (2 frames) and longer (p<0.05, binomial cumulative distribution function; Fig 2C). Individual performance never reached ceiling; the highest-performing animal achieved a maximum of 95% correct at the 100 ms target duration. We suspect that performance would not substantially improve given larger sampling times, as in many studies, rats tend to have relatively high error rates even on the easiest trials of detection or discrimination tasks [33, 43]. For all rats, performance significantly increased with target duration (Fig 2C; p_A_<0.0001, χ^2^ (7, N = 1243) = 41.81; p_B_<0.05, χ^2^ (3, N = 829) = 10.40; p_C_<0.0001, χ^2^ (7, N = 2380) = 112.68; p_D_<0.0001, χ^2^ (7, N = 2619) = 101.18; p_E_<0.0001, χ^2^ (7, N = 1856) = 57.13; p_F_<0.0001, χ^2^ (7, N = 1529) = 93.74; p_G_<0.0001, χ^2^ (8, N = 1943) = 40.98; Chi-square goodness of fit).

### Experiment 2: Visual masking does not reduce orientation discrimination performance

**Fig 3:**
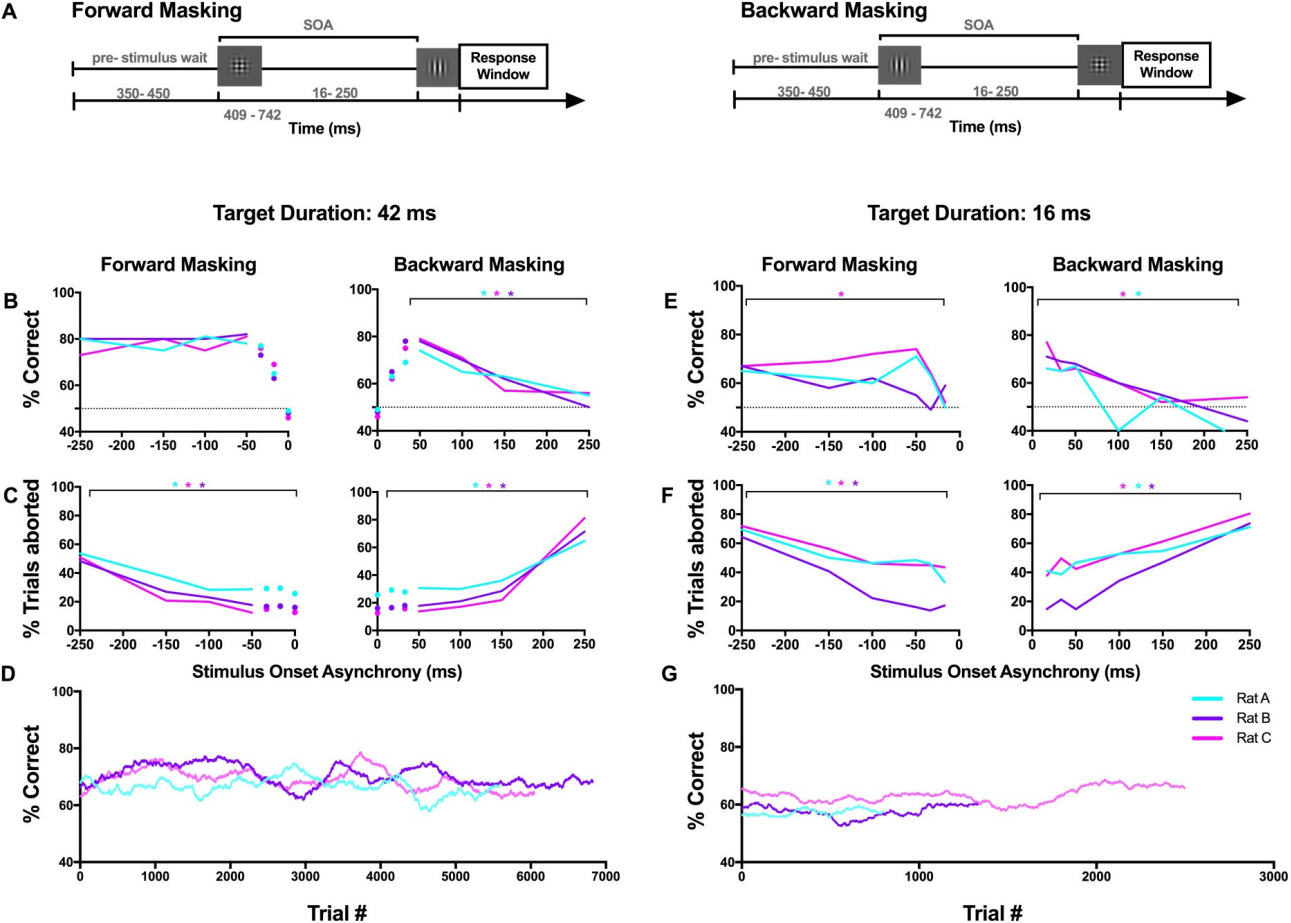
Spatially overlapping masks do not affect rat orientation discrimination performance. (A) Structure of forward (left) and backward (right) masking trials. (B) Orientation discrimination performance and (C) percentage of aborted trials for three animals is shown for the 42 ms target duration. (D) Overall task performance for each rat plotted as a 500-trial running average during data collection. Rats performed roughly 500 trials per day. (E-G) same as (B-D) for the 16 ms target duration. At an SOA of 0 only the mask was presented and rewards were given randomly; therefore, these values reflect how often the animal was rewarded. The dotted line indicates chance performance. In (E) the temporally overlapping stimulus onset asynchronies (SOA) where target duration was reduced, are presented separately from the remainder of the curve. For rat A, the 42 ms target duration data represents a summary of performance across 244 trials/SOA and 85 sessions, the 16 ms data 96 trials/SOA and 21 sessions (Rat B: 42 ms—229 trials/SOA, 66 sessions; 16ms—142 trials/SOA; 16 sessions. Rat C: 42 ms—265 trials/SOA, 65 sessions; 16 ms—211 trials/SOA, 32 sessions).

In Experiment 2, we examined how a spatially overlapping mask affected rat discrimination performance for a 42 ms (Fig 3B–3D) and 16 ms target (Fig 3E–3G) in both forward and backward masking trials (Fig 3A). In each trial, a mask was presented at one of 13 stimulus onset asynchronies (SOA) relative to the target. Below, we separately describe the results for forward, and backward masking trials. Note that at an SOA of 0, only a mask stimulus was presented, allowing us to check for response bias. On these trials, animals showed no significant bias in how often they selected the left response port (%left_A_ = 54.3; %left_B_ = 50.7; %left_C_ = 50.0. Binomial cumulative distribution tests, p>0.05).

#### Forward masking

Under forward masking conditions, our neuronal decoder predicted that rat performance would decrease towards the shorter SOAs. In the 42 ms target condition we found that discrimination performance for SOAs less than -33 ms was strongly impaired compared to when longer SOAs were used (Fig 3B). While reminiscent of commonly reported masking phenomena, unfortunately, this finding was complicated by the fact that at these SOAs, the duration of the target was reduced by the presence of the mask, and in experiment 1, we found that performance was impacted by the target duration. Therefore, it was impossible to determine if the lower discrimination performance was the result of changing target duration alone or if the presence of a mask immediately adjacent to the target was additionally impairing target perception. We therefore only analysed the effect of SOA on discrimination performance over SOAs where the target duration was consistently 42 ms (|SOA|≥50 ms). In these cases, we found rat performance was not altered across SOA (Fig 3B-left; p_A_ = 0.310, X^2^ (3, N = 1440) = 3.582; p_B_ = 0.890, X^2^ (3, N = 1541) = 0.628; p_C_ = 0.477, X^2^ (3, N = 969) = 2.492; Chi-square goodness of fit).

To better determine if perceptual masking was present at the short SOAs (<50 ms), we ran 22–32 sessions/animal using a 16 ms target, so that target duration was consistent across all SOAs. We found that discrimination performance significantly decreased towards the shorter SOAs for only one animal; the rat with the highest overall performance and that had completed the largest number of trials (Fig 3E-left; p_A_ = 0.117, X^2^ (5, N = 500) = 8.802; p_B_ = 0.326, X^2^ (5, N = 472) = 5.806; p_C_<0.0001, X^2^ (5, N = 998) = 27.383; Chi-square goodness of fit). The two animals that did not display any significant effects of masking also showed a higher target duration threshold in experiment 1, suggesting they were performing at the threshold of their capabilities, which was determined in the absence of a mask. It is likely that the introduction of a mask reduced overall performance and therefore compromised our ability to observe perceptual masking in these animals. Altogether this suggests that even if perceptual masking was occurring, performance levels were too close to chance to reveal any significant effects across SOA in two of the animals.

Given that rats tend to respond impulsively on some trials, we also considered how the proportion of aborted trials varied across SOA. As the duration of the trial covaried with SOA, we expected that there might be a greater proportion of aborted trials at the longer SOAs. This was true for all animals and both target duration conditions (16 ms: p_A_<0.0001, X^2^ (5, N = 1130) = 70.224; p_B_<0.0001, X^2^ (5, N = 974) = 299.534; p_C_<0.0001, X^2^ (5, N = 2603) = 269.178; 42 ms: p_A_<0.0001, X^2^ (5, N = 4963) = 215.598; p_B_<0.0001, X^2^ (5, N = 5357) = 379.025; p_C_<0.0001, X^2^ (5, N = 4555) = 597.35; Chi-square goodness of fit).

#### Backward masking

Under spatially overlapping backward masking conditions, the neuronal decoder predicted that rat performance would decrease with SOA. In the 42 ms target condition, across the the SOAs where target duration was consistent (SOA: 50–250 ms), we found that performance increased significantly towards the short SOAs for all rats (Fig 3B-right; p_A_<0.0001, X^2^ (3, N = 1380) = 30.244; p_B_<0.0001, X^2^ (3, N = 1329) = 60.142; p_C_<0.0001, X^2^ (3, N = 819) = 41.314; Chi-square goodness of fit). Similarly, in the 16 ms target condition, performance significantly increased towards the short SOAs for two of three rats (Fig 3E-right; p_A_<0.0001, X^2^ (5, N = 484) = 28.697; p_B_ = 0.069, X^2^ (5, N = 364) = 10.234; p_C_<0.0001, X^2^ (5, N = 1881) = 27.185; Chi-square goodness of fit). Given that this trend is atypical of visual masking [42], it is likely that these changes in performance across SOA reflect behavioural aspects other than perceptual masking, such as changes in attention or impulsivity. When we considered aborted trials, we found there was an increased proportion at the SOAs where discrimination performance was impaired (Fig 3C-right; 42 ms: p_A_<0.0001, X^2^ (5, N = 5048) = 319.671; p_B_<0.0001, X^2^ (5, N = 5568) = 646.612; p_C_<0.0001, X^2^ (5, N = 5045) = 951.228; Fig 3F-right; 16 ms: p_A_<0.0001, X^2^ (5, N = 1203) = 108.467; p_B_<0.0001, X^2^ (5, N = 1078) = 379.522; p_C_<0.0001, X^2^ (5, N = 2842) = 415.1; Chi-square goodness of fit). This may reflect an interaction between impulsive behaviour and discrimination performance in this task. However, given that similar increases in the proportion of aborted trials did not have a detrimental effect on discrimination performance at long forward masking SOAs, it seems likely that the order of stimuli plays an important role. For example, the differences in behaviour between forward and backward masking conditions might reflect an imbalance in the working memory load as responses must be withheld in the backward masking task until after the mask has been presented.

Throughout the data collection phase we found that performance levels were stable for all rats (Fig 3D and 3G). Thus, it is unlikely that the unusual backward masking trends were the result of insufficient time to learn the task. However, as the forward and backward masking trials were collected simultaneously, it is possible that the animals learnt to respond in a way that favoured forward masking trials; i.e. always ignore the first stimulus and respond to the second. This problem could be easily avoided if forward and backward masking data were collected in discrete sessions (See Experiment 3).

### Experiment 2: Rat response times change with trial duration

To better understand how rat behaviour was affected by our task parameters, we calculated response times for the 42 ms target condition based on when the rats exited the central port relative to both target and mask onset. Given the possibility of rats making decisions that were not influenced by the stimulus orientation, we were particularly interested to determine whether the rats responded at a fixed time following the appearance of the informative target stimulus, or whether their responses were simply locked to the appearance of the first stimulus. Note that in all trials, rats had 16.6 seconds to initiate their response, so there was no penalty for long response times and no explicit incentive for short response times. Response times were measured in all trials, including aborted trials and correction trials.

**Fig 4:**
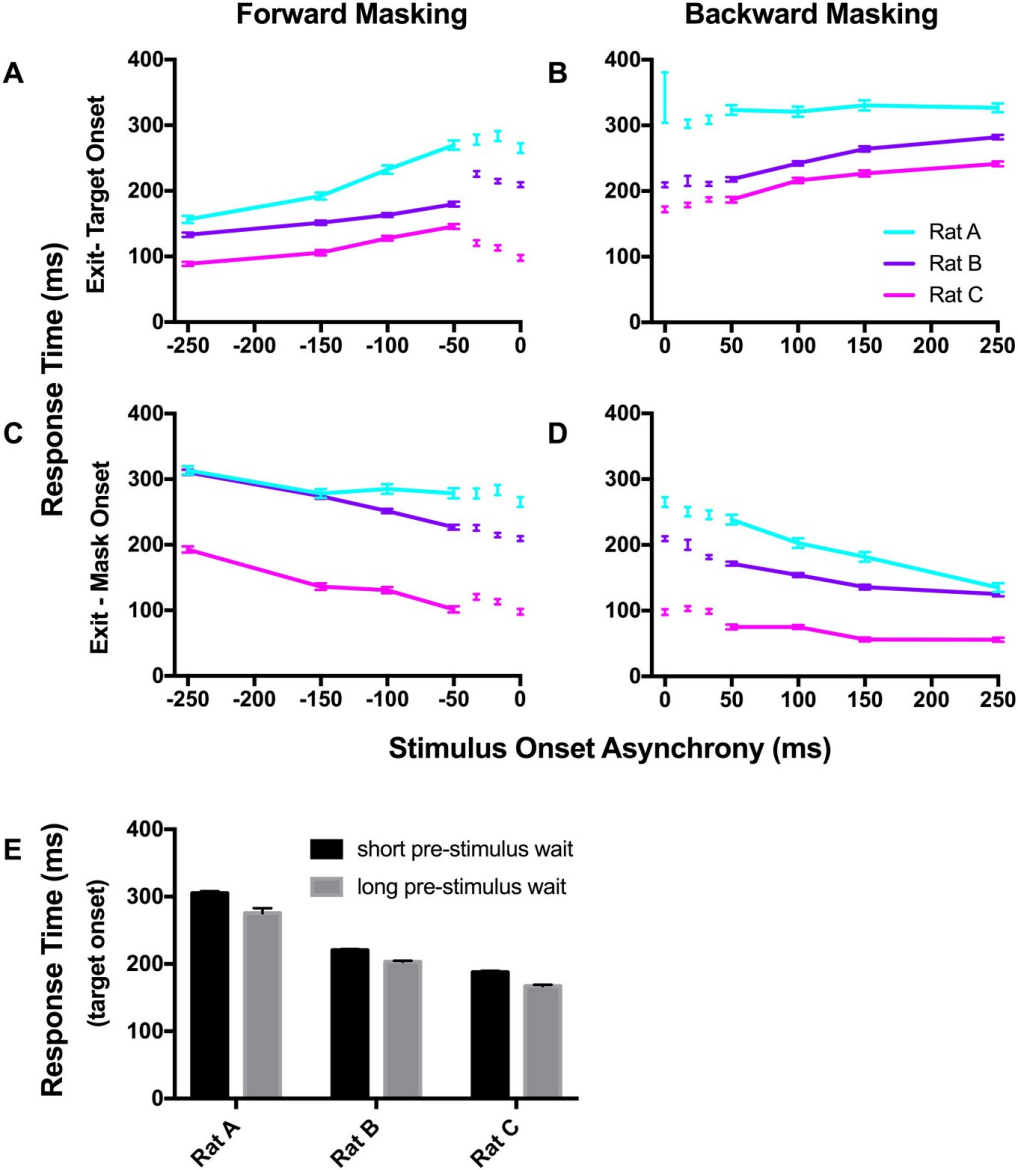
Rat response times are influenced by trial duration but not by visual masking. Response times were measured for the 42 ms target condition from the (A, B) target onset and (C, D) mask onset to the time that the rat exited the central sensor. For each rat, all response times are represented as mean (SE). Response times measured at the temporally overlapping SOAs, where target duration was truncated, are presented separate from the remainder of the curve. At a SOA of 0, only a mask stimulus was presented. (E) rat response times measured from the target onset divided according to the duration of the pre-stimulus hold, collapsed across SOA (pre-stimulus hold: 350–400 & 400–450 ms). For rat A, data measured from the target onset represents a summary of performance across an average of 453 forward (496 backward) trials/SOA and 85 sessions, mask onset data 551 (517) trials/SOA and 85 sessions. Rat B–target onset: 620 (743) trials/SOA; 66 sessions, mask onset: 690 (632) trials/SOA; 66 sessions. Rat C–target onset: 341 (419) trials/SOA; 65 sessions, mask onset: 641 (572) trials/SOA; 65 sessions.

In forward masking trials, if the rats responded at a fixed duration following the target stimulus then response times measured from target onset should be unaffected by SOA, i.e. a flat line in Fig 4A. Instead response times significantly increased as the SOA shortened (p_A_<0.0001, F_3,1823_ = 65.53; p_B_<0.0001, F_3,2535_ = 32.95; p_C_<0.0001, F_3,1259_ = 42.98; one-way ANOVA) and the response times were significantly different for all SOA comparisons (p_A_<0.001, p_B_<0.01, p_C_<0.01; Tukey's multiple comparisons test).

On the other hand, if the rats responded at a fixed duration following the mask, then we would expect response times measured from the onset of the mask to remain constant across varying SOAs (Fig 4C). Again, this was not the case, the response times significantly decreased with SOA for all animals (p_A_<0.001, F_3,2314_ = 6.14; p_B_<0.0001, F_3,3008_ = 83.21; p_C_<0.0001, F_3,2634_ = 63.11; one-way ANOVA). Post hoc analyses revealed that response times were significantly different for all SOA comparisons in Rat B, for all except -100 vs -150 ms in Rat C, and for only the longest SOA of -250 ms in Rat A (p_A_<0.05, p_B_<0.001, p_C_<0.001; Tukey’s multiple comparisons test).

Similarly, in backward masking we found that rats did not respond at a fixed duration following the target or mask stimulus. Response times measured from the target onset decreased with SOA for two rats (Fig 4B; p_B_<0.0001, F_3,3366_ = 55.26; p_C_<0.0001, F_3,1797_ = 25.19; one-way ANOVA), with response times being significantly different for all SOA comparisons in rat B and all except two (100 vs 150 ms and 100 vs 250 ms) in rat C (p_B_<0.01, p_C_<0.001; Tukey’s multiple comparisons test). The response times for rat A remained consistent across SOA (p_A_ = 0.8345, F_3,2110_ = 0.287; one-way ANOVA).

Finally, when measuring the response times from the mask onset, we saw a significant increase towards the shorter SOAs for all rats (Fig 4D; p_A_<0.0001, F_3,2074_ = 35.91; p_B_<0.0001, F_3,2605_ = 34.94; p_C_<0.0001, F_3,2169_ = 10.86; one-way ANOVA). The response times were significantly different for most SOA comparisons (p_A_<0.01, p_B_<0.01, p_C_<0.001; Tukey’s multiple comparisons test), with a few non-systematic exceptions (rat A—100 vs. 150 ms; rat B—150 vs. 250 ms; rat C—50 vs 100 ms and 150 vs 250 ms).

Collectively, our results indicate that the rats did not respond at a fixed duration following the onset of either the target or mask stimulus. Interestingly the trends in response times were consistent between forward and backward masking conditions when considering the order of stimuli; the response times increased towards longer SOAs when measured from the first stimulus onset and decreased towards the longer SOAs when measured from the last stimulus onset. This suggests that the rats began planning their response as soon as the trial began regardless of when the target appeared and therefore response times were most affected by the duration of the trial. In line with this idea, when response times were separated into two groups according to the duration of the pre-stimulus hold, we found that trials with longer pre-stimulus durations were associated with shorter response times for all rats (Fig 4E). This was found to be significant across all rats (p_A_<0.0001, t_2864_ = 4.675; p_B_<0.0001, t_4310_ = 5.275; p_C_<0.0001, t_2403_ = 6.398; paired t-test).

### Experiment 3: Backward masking does not impair rat orientation discrimination

**Fig 5:**
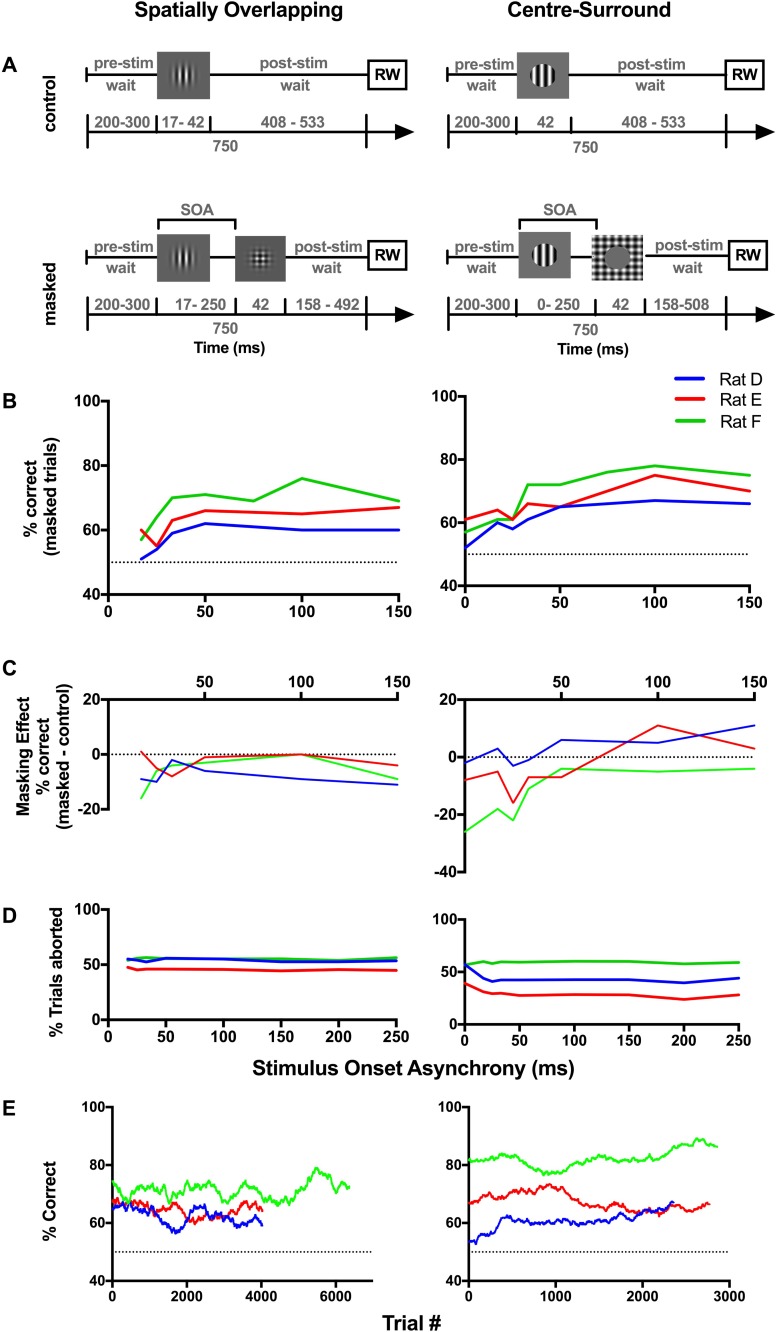
Spatially overlapping and centre-surround backward masking does not impair discrimination performance in rats. (A) Schematic of trial structure for control (top) and masked (bottom) trials using spatially overlapping (left) and centre-surround (right) stimuli. RW indicates the response window. (B) Orientation discrimination performance of three animals. The dotted line at 50% correct represents the chance performance level. (C) The effect of the mask, measured as the difference in performance between control and masked trials. The dotted line at 0% indicates when performance is equal across control and masked trials. (D) Percent aborted trials. (E) Overall task performance for each rat plotted as a 500-trial running average during data collection. Rats performed roughly 500 trials per day. For rat D, spatially overlapping data represents a summary of performance across an average of 378 trials/SOA and 63 sessions, centre-surround data 300 trials/SOA and 52 sessions (Rat E–SO: 370 trials/SOA; 77 sessions, CS: 320 trials/SOA; 52 sessions. Rat F–SO: 602 trials/SOA; 64 sessions, CS: 1083 trials/SOA; 112 sessions).

In experiment 2 we did not find convincing evidence of backward masking in rats, but our experimental design was limited as it appeared to be subject to the influence of impulsive behaviour, which may have impaired our ability to observe perceptual masking. To further investigate whether visual masking might affect rat perception, in experiment 3 we used 100% contrast masks that were presented either overlapping or surrounding the target location. Three new rats were trained to complete sessions with control and masked trials randomly interspersed (Fig 5A). Importantly, we focused on backward masking and introduced a fixed 750 ms trial duration by adding a post-stimulus waiting period, which removed the possible problems associated with impulsivity that may have affected the long SOA trials in Experiment 2. We also introduced control trials in which no mask was presented and target duration was matched to that in masked trials; in the spatially overlapping condition, these required truncating the target duration to 16 or 33 ms.

In experiment 2, we were concerned that the variation in trial duration across SOA was interacting with impulsivity resulting in a larger proportion of aborted trials and possibly impaired performance at the long SOAs. This idea is supported by our findings in fixed duration trials that the proportion of aborted trials was stable across SOA for both spatially overlapping (Fig 5D-left; p_D_ = 0.558, X^2^ (7, N = 7328) = 5.842; p_E_ = 0.327, X^2^ (7, N = 5706) = 75.45; p_F_ = 0.398, X^2^ (8, N = 15352) = 8.378; Chi-square) and centre-surround configurations (Fig 5D-right; p_D_ = 0.032, X^2^ (7, N = 3793) = 16.787; p_E_ = 0.068, X^2^ (7, N = 2933) = 14.56; p_F_ = 0.059, X^2^ (9, N = 25486) = 8.378; Chi-square). Furthermore, instead of increasing towards the longer SOAs, discrimination performance reached a plateau at an SOA of 100–150 ms. We therefore restricted our analyses to only consider SOAs up to 150 ms. Throughout data collection, performance levels were stable (Fig 5E).

In the *spatially overlapping* condition, we expected that if rats were affected by visual masking, their performance in masked trials would be similar to that of control trials at long SOAs, but would be impaired relative to control performance at shorter SOAs. Instead, for all rats, performance was consistently lower in the masked trials across all SOAs (Fig 5B and 5C-left. p_D_<0.0001, X^2^ (5, N = 1810) = 57.154; p_E_<0.0001, X^2^ (5, N = 1868) = 109.720; p_F_<0.0001, X^2^ (6, N = 3380) = 721.9209; Chi-square). Our results indicate that the presence of a mask reduces orientation discrimination performance even at long SOAs, but that the reduction occurs in a manner that is independent of SOA and is thus uncharacteristic of visual masking.

In the *centre-surround* configuration, the mask was a full screen plaid with an aperture matching the size and location of the target. Therefore, the target remained visible for 42 ms for all SOAs, allowing us to assess the effects of a mask at short SOAs without confounding changes in target duration. The results from our neuronal decoder predicted that rat performance would be impaired at the short SOAs when using a surround mask. Again, we found that discrimination performance was significantly different between control and masked conditions for all SOAs, not just the short SOAs (Fig 5B and 5C-right; p_D_<0.01, X^2^ (6, N = 1207) = 20.50; p_E_<0.0001, X^2^ (6, N = 1285) = 59.68; p_F_<0.0001, X^2^ (7, N = 4341) = 522.19; Chi-square). Although performance tended to decrease towards an SOA of 0 for all animals in the masked trials, strangely, masked performance was sometimes better than control performance in two of the animals. However, the performance of Rat F agreed with our broad predictions for visual masking in rats, with the difference between masked and control performance systematically lower at short SOAs.

Together, our results suggest that the mere presence of a mask stimulus impairs rat performance regardless of SOA. Further, the effect of SOA on rat performance only followed the trends predicted by our neuronal decoder in some animals. This may be because the overall performance levels were too low to consistently reveal perceptual masking across animals, rather than because our stimuli were not actually capable of producing perceptual deficits.

### Our visual masking stimuli impairs human orientation discrimination at short stimulus onset asynchronies

**Fig 6:**
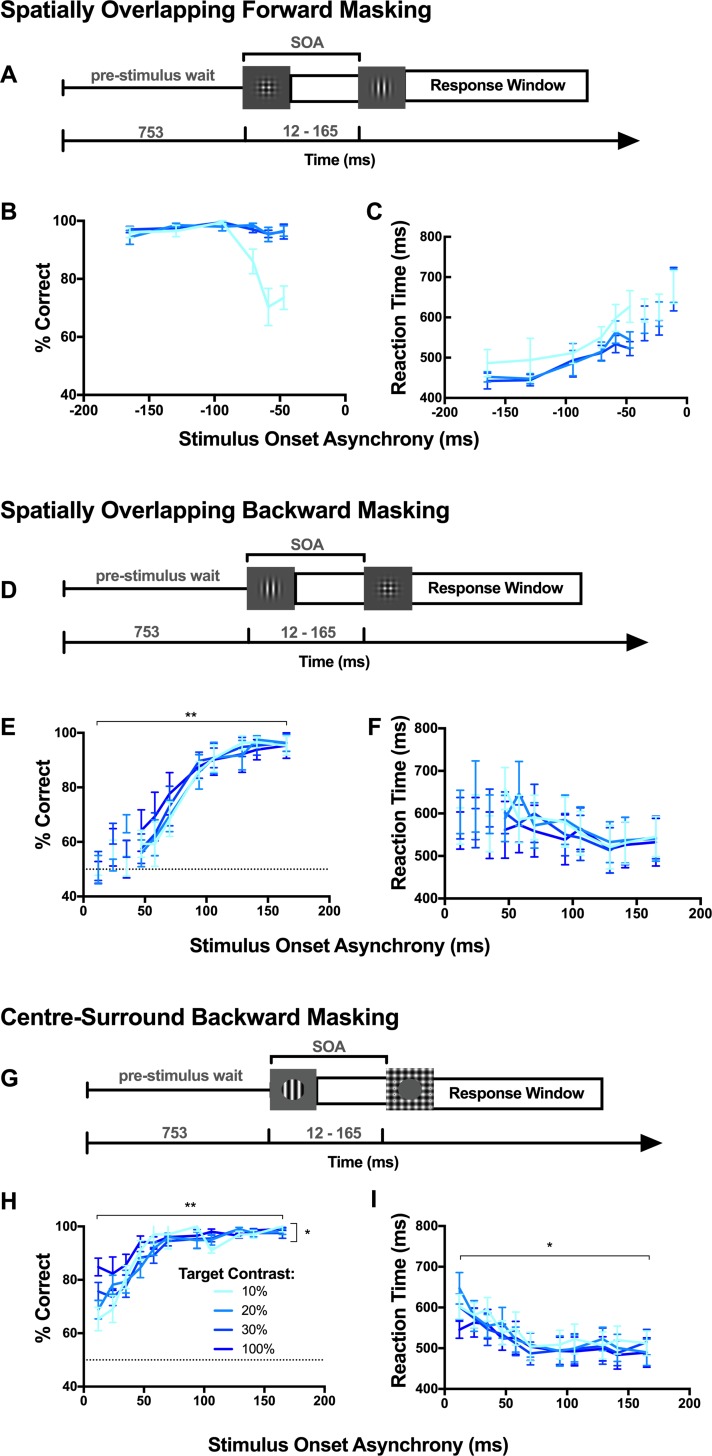
Human orientation discrimination and reaction times are influenced by mask stimuli at short stimulus onset asynchronies (SOAs). (A) Schematic of trial structure for spatially overlapping forward masking. (B) Orientation discrimination performance across six participants (mean, SE) for three target contrasts (10,20 and 30%). The dotted line represents chance performance. (C) Mean (SE) reaction times, measured from target onset until keypress response. Performance and reaction times for temporally overlapping SOAs, where the target duration was truncated, are presented separately from the remainder of the curve. (D-F) same as (A-C) for spatially overlapping backward masking. (G-I) Same as for (A-C) for centre-surround backward masking. (**) p<0.0001; (*) p<0.001.

We did not find consistent evidence of perceptual masking in rats, a phenomenon that has been repeatedly observed in humans and non-human primates [1, 44]. To determine how our masking stimuli affected human perception, we measured the effects of masking on human orientation discrimination performance, using stimuli similar to those used in our rat experiments (Fig 6A, 6D and 6G). In separate testing sessions, we presented stimuli with spatially overlapping or centre-surround configurations.

For *spatially overlapping* stimuli, we collected both forward and backward masking data across separate testing blocks. All temporally overlapping SOAs, where the target duration was shortened, were excluded from statistical analyses. Under forward masking conditions, performance increased with longer SOAs, saturating near 100% correct for SOAs above 100 ms, regardless of target contrast (Fig 6B). Using a two-way ANOVA of arcsine transformed performance (SOA x target contrast), we found performance averaged across participants was significantly affected by SOA (p_SOA_<0.0001, F_5, 25_ = 8.75) and contrast (p_Contrast_<0.0001, F_2, 10_ = 27.36), and that there was a significant interaction between the factors (p_SOA x Contrast_<0.0001, F_10, 50_ = 7.62).

Similar to forward masking conditions, backward masking performance increased with SOA, saturating near 100% correct for the majority of participants at SOAs of 130 ms and longer (Fig 6E). This trend remained relatively robust regardless of the target contrast. Using two-way ANOVA (SOA x target contrast), we found performance averaged across participants was affected by SOA (p_SOA_<0.0001, F_7, 35_ = 23.29). However, there was no significant effect of target contrast (p_Contrast_ = 0.20, F_3, 15_ = 1.73), nor was there any significant interaction between target contrast and SOA (p_SOA x Contrast_ = 0.87, F_21, 105_ = 0.65). Post hoc analyses revealed performance was significantly lower at the four shortest SOAs (47–94 ms) for all target contrasts when compared to the longest SOA (p<0.05; ANOVA; Dunnett’s multiple comparison *post hoc*).

Despite the lack of explicit instructions regarding response timing, under *spatially overlapping* conditions, forward masking reaction times were significantly affected by both SOA and target contrast, but there was no significant interaction between factors (Fig 6C; p_SOA_<0.0001, F_5, 90_ = 8.23; p_Contrast_<0.01, F_2, 90_ = 5.92; p_SOA x Contrast_ = 0.98, F_10, 90_ = 0.30; two-way ANOVA). Post hoc analyses revealed that at 10% target contrast reaction times were longer for two SOAs (-47 & -58 ms), and at 20% contrast for one SOA (-58 ms) when compared to the longest SOA (p<0.05; ANOVA; Dunnett’s multiple comparison *post hoc*). On the other hand, reaction times for spatially overlapping backward masking were not significantly affected by SOA or target contrast, and there was no significant interaction between the two factors (Fig 6F; p_SOA_ = 0.33, F_7, 160_ = 1.16; p_Contrast_ = 0.78, F_3, 160_ = 0.36; p_SOA x Contrast_>0.99, F_21, 160_ = 0.13; two-way ANOVA).

Using *centre-surround* backward masking stimuli, discrimination performance for each participant increased with SOA and saturated at SOAs of 58 ms and longer (Fig 6H). Performance averaged across participants was found to significantly increase with SOA (p_SOA_<0.0001, F_10, 50_ = 29.67; two-way ANOVA) with performance significantly lower at the five shortest SOAs (12–58 ms) for target contrasts 20, 30 and 100%, and at the six shortest SOAs (12–70 ms) for a 10% target contrast when compared with the longest SOA (165 ms; p<0.05; ANOVA; Dunnett’s multiple comparison *post hoc*). There was also a significant main effect of target contrast on orientation discrimination, where orientation discrimination performance improved as target contrast increased (p_Contrast_<0.01, F_3, 15_ = 9.36; two-way ANOVA). Significant interaction effects revealed this effect of target contrast was largest at the short SOAs (p_SOA x Contrast_<0.05, F_30, 150_ = 1.96; two-way ANOVA). At 10% contrast, discrimination performance was lower than that of 100% contrast for the five shortest SOAs (12–58 ms). At 20% contrast the drop in performance was only significant for two short SOAs (12 and 47 ms) and at 30% contrast only for a single SOA (24 ms; p<0.05; ANOVA; Tukey’s multiple comparison *post hoc*).

Under *centre-surround* conditions, the effect of SOA on reaction times averaged across participants was significant (Fig 6I; p<0.0001, F_10, 220_ = 4.60; two-way ANOVA), but there were no significant effects of target contrast or any interaction (p_Contrast_ = 0.34, F_3, 220_ = 1.13; p_SOA x Contrast_>0.99, F_30, 220_ = 0.21; two-way ANOVA).

Our data demonstrate that, regardless of the spatial configuration of visual masking stimuli, human orientation discrimination performance is impaired at short stimulus onset asynchronies. Although this was similar to the predictions of our neuronal decoder, we did not consistently observe these trends in rat behaviour. We also found that at the SOAs where human performance was impaired, the reaction times were longer. Collectively this suggests that human reaction times increase when there is uncertainty about the target orientation, rather than relating to the trial duration as was the case in rats.

## Discussion

We did not find any consistent evidence of visual masking in rats, even though our analysis of the activity of populations of neurons from visual cortex of anaesthetized rats indicated the presence of masking at short SOAs. Further, we used SOAs where perception is known to be impaired in humans, and stimuli similar to those used in the rat task were capable of producing perceptual deficits at these SOAs in humans. Critically, all rats could reliably discriminate the orientation of a Gabor target in a discrimination task. However, in masked trials, the rats’ discrimination performance was reduced and only exhibited the temporal profile characteristic of visual masking in some animals. Altogether, our results indicate that perceptual masking may occur in rats, but that it is difficult to observe due to behavioural and visual limitations. Below we discuss: 1) the discrepancy between neuronal and perceptual results in rats; 2) the importance of task design; 3) the differences in rat and human behaviour; 4) our results in relation to the most prominent theories of visual masking; and 5) the temporal acuity of rat vision.

Our perceptual findings in rats were not always consistent with the pattern of reduction in orientation discriminability observed in V1 neurons recorded in anaesthetized rats [2]. Our neuronal data revealed that firing rates and orientation discriminability were impaired at short SOAs (|SOA|<100 ms) [2]. For all stimulus conditions, our neuronal population decoder predicted that the ability of rats to discriminate target orientation would decrease towards a SOA of zero, a typical trend observed in visual masking [42]. However, rat perceptual performance only followed these trends in a limited number of animals and experimental circumstances. It is possible that perceptual masking is absent in rats, despite the fact that similar stimuli can alter neuronal processing in V1. It could be that activity in V1 was not sufficiently altered to disrupt rat perception or alternatively that V1 does not play a direct role in informing rat behaviour. Certainly, in the case of object discrimination, neuronal activity better reflects rat behaviour in later stages of visual processing when compared to early areas like V1 [45]. Furthermore, neuronal responses in lateral extrastriate areas become more tolerant to image transformations as visual processing progresses [30]. Thus, it might be that higher order areas are more tolerant to the effects of visual masking and better account for rat orientation discrimination performance. It is also possible that the small differences in the stimuli used for our neuronal and perceptual study (square wave versus sine wave) were sufficient to remove the effects of visual masking in our behavioural task. However, given that two animals did show behavioural trends that were congruent with perceptual masking, and that these animals performed more trials with a higher overall performance, it is more likely that perceptual masking does occur in rats, but that the effect size is too small to be revealed consistently across rats performing a task with a relatively high lapse rate. A recent study also suggests that inter-individual differences in perceptual strategy may occur, making it even more challenging to reveal small effects in both individual animals and at the population level [46]. Because of this inter-individual variability and our relatively small number of animals, we cannot exclude the possibility that with a larger cohort, including more high-performing rats, visual masking may manifest more strongly. However, it is important to note that the stimulus manipulations that increase the strength of visual masking (shorter target duration, lower target contrast), also lead to lower discrimination performance overall. Thus, the manipulations necessary to quantify masking, if it occurs, are a trade-off working directly against the manipulations that allow us to measure psychophysical performance in the first place. Ultimately, if perceptual masking cannot be reliably observed in rat behaviour then a rat model may not be suitable for investigating the effects of visual masking in a discrimination task.

Task design can have a large impact on animal behaviour, and any resulting models of animal perception [47]. The results of our study suggest an orientation discrimination task may not be ideal for visual masking research in rats, however, we cannot rule out the possibility that perceptual masking might be observed in a different type of task. A number of visual masking studies have implemented a detection task [5, 6], which is arguably a simpler task to perform. In a detection task, it may be possible to maintain higher performance using shorter target durations, thereby improving both the sensitivity of the task for measuring the effects of masking and increasing the likelihood of visual masking occurring. However, a visual masking detection task would come with its own difficulties, as the rats would need to be able to respond to one type of visual stimulus while ignoring another of similar appearance. In a study investigating the effects of flanking Gabors on target Gabor detection, Long-Evans rats were required to respond to a target while ignoring synchronously presented flankers (similar to our centre-surround task with an SOA of 0 ms) [43]. In this task, the presence of flankers impaired target detection, but also biased the animals to respond, even in the absence of a target [43]. It is therefore likely that rat performance would be similarly biased by the presence of a mask. Altogether this implies that essential aspects of visual masking, such as the inclusion of multiple, briefly presented stimuli, limit the performance of rats and thus our ability to observe perceptual masking in rats.

In a similar task to that used in rats, we found that human perception was impaired in a monotonic trend that is typical of visual masking. In the spatially overlapping condition, perception was impaired at short SOAs for both forward and backward masking conditions (|SOA| < 100 ms). This was consistent for all target contrasts in backward masking, but only for the 10% contrast in forward masking, with performance at the higher contrasts reaching ceiling performance earlier (SOA>-50 ms). In rats, where target contrast was always 100%, we only saw evidence of perceptual masking under forward masking conditions and for only one rat. Similarly, for centre-surround backward masking, human performance was impaired up to SOAs of 70 ms, a trend that was similar to the performance of one rat. These inconsistencies between species could reflect differences in task performance capabilities, in the strength of the masking effect, or in the temporal sensitivity and dynamics of visual masking. In an orientation discrimination task, human performance is both high, and stable. Therefore, any factor that yields a small, reliable change in performance will be easily observable. In contrast, rat performance is lower, and inherently more variable, so any factor yielding a change in performance would need to produce a large change to be observed. In addition, it is possible that the strength of masking was different between species. Although we attempted to keep stimuli as similar as possible between human and rat experiments, the large differences in human and rat visual abilities, for example in spatial acuity and receptive field size, meant that it was necessary to alter some aspects of the stimuli, namely the size of the target stimulus [26, 48–51]. Given that the strength of the mask is highly dependent on stimulus properties, such as size, contrast and duration, it is possible that the strength of the mask was weaker in the rat experiments and therefore only occasionally evident in the animal behaviour [52, 53]. Finally, if the temporal dynamics of visual masking were different between species, it could be that we failed to observe consistent effects of visual masking in rats because we did not sample the relevant SOAs. However, this seems unlikely given that the predictions using rat neuronal responses indicate similar trends over similar SOAs to that of the human perceptual data.

The perceptual effects of visual masking arise through a combination of neuronal mechanisms acting across multiple regions throughout the visual processing hierarchy [2, 13, 15, 20, 54]. The specific combination of these mechanisms is thought to vary depending on the properties of the stimuli, in particular the spatial configuration of the target and mask [1]. Although there are many proposed mechanisms, the majority of neuronal theories explaining perceptual masking can be grouped into two broad categories; neural interruption and neural integration [55]. The first of these theories proposes that neuronal processing of the target is abandoned at the arrival of activity evoked by the mask. This is a favoured explanation for U-shaped visual masking, where the greatest impairment in perception occurs at an intermediate SOA (~ 50–100 ms). However, neural interruption is contingent on the mask being presented after the target in time and is therefore incapable of explaining forward or common-onset (SOA = 0) masking [56]. Given that the impairments in human perception, and the predictions of our neuronal decoder, always decreased monotonically towards a SOA of 0, it seems unlikely that neural interruption was contributing to our results.

On the other hand, neural integration proposes that, due to temporal processing limits of the visual system, at short SOAs the target and mask are combined in one ‘perceptual window’ resulting in the perception of a single, fused image [10, 57]. As a result, the visibility and perception of the target is reduced. Most researchers agree that neural integration is involved in monotonic visual masking trends, which we observed in our human participants, our neuronal population decoding, and in some of the rat behaviour [6, 55, 58]. If the size of this ‘perceptual window’ over which information is integrated were smaller in rats than in humans, then it might explain why perceptual masking was so rarely observed. To the best of our knowledge, the temporal acuity of rat vision has only been defined through critical fusion frequency (CFF), the frequency at which a flickering light appears to become continuous [59]. In hooded-rats, CFF lies between 15–20 Hz (5.5 cd/m^2^ luminance), suggesting that their visual perception may be integrated across windows of 50–66 ms [60]. This is worse than the temporal acuity of humans, where the CFF lies around 50–90 Hz, predicting integration windows of 11–20 ms [60, 61]. Although this would suggest that the effects of perceptual masking in rats should extend to longer SOAs than in humans, it should be noted that if neural integration were the principle mechanism acting in monotonic visual masking, the CFF could not be a good measure of the ‘perceptual window’ size, as a 50–90 Hz CFF would suggest that visual masking in humans would only ever occur at SOAs shorter than 20 ms, which we have shown to be false.

Numerous rodent studies have stressed the importance of acuity considerations in visual task design [26, 62, 63], however, these studies have only addressed spatial and not temporal aspects of visual acuity. It is critical to consider the temporal acuity of animals in any behavioural study to ensure that the duration of the stimulus is appropriate for the animals to perform the task correctly. While the CFF provides some information about temporal acuity, it does not directly address the minimum duration of a stimulus that can be detected or discriminated. In Long Evans rats, the ability to discriminate the direction of motion in moving dots with 85% coherence was shown to increase with stimulus duration and plateau at 75% correct for durations of 200 ms and longer [64]. However, the effect of stimulus duration has never been addressed for static stimuli. Here we identified a threshold stimulus duration at which rats can reliably discriminate stimulus features such as orientation: all rats performed significantly above chance for durations of 16 ms or longer. However, for a task that does not necessitate constraints on stimulus duration, our data suggests durations of 60 ms or greater would be ideal to maintain high performance levels. Here, we used 16 and 42 ms because we anticipated that longer target durations would be more difficult to mask.

In spite of controls in experimental design and stimulus properties, we found that visual masking did not consistently affect rat performance in the same way that it affected humans, nor did rat performance follow the patterns predicted from neuronal responses collected in V1 of anaesthetized rats. The introduction of a mask stimulus reduced overall task performance and compromised the sensitivity of the data to reveal any effects of perceptual masking. Unfortunately, the parameter manipulations that would increase the size of the masking effect, also reduce performance. Thus, it may be difficult to consistently observe perceptual masking in rats. Our results indicate there may be limitations for the applications of rat behaviour in the study of visual masking and perception. While rats may still provide some significant advantages for investigating aspects of visual processing, there may be some significant limitations in the types of tasks that they can perform with adequate performance levels.
